# A Rare Diagnosis of Paraesthesia in the Pediatric Age Group: Unmasking the Male Factor

**DOI:** 10.7759/cureus.46047

**Published:** 2023-09-27

**Authors:** Yashasvi Anand, Sanket S Bakshi, Swaroopa Chakole

**Affiliations:** 1 Department of Medicine, Jawaharlal Nehru Medical College, Datta Meghe Institute of Higher Education and Research, Wardha, IND; 2 Department of Community Medicine, Jawaharlal Nehru Medical College, Datta Meghe Institute of Higher Education and Research, Wardha, IND

**Keywords:** myelopathy, laminectomies, paraesthesia, cyst, extradural

## Abstract

Spinal extradural arachnoid cysts are an uncommon condition and their exact causative triggers are still unclear. They appear to be extradural arachnoid outpouchings that connect to the intraspinal subarachnoid region via a little dura defect. These cysts are most commonly seen in the thoracic spine, followed by the lumbosacral junction. Compression of the spinal cord or nerve roots leads to the development of the symptoms. The most morbid symptom associated with these compressing extradural cysts is paresthesia. Numerous theories have been proposed about their origins, and the related conditions include spinal trauma, spina bifida, and the lymphedema-distichiasis syndrome. Their position in the spine influences the symptoms manifested. The diagnosis is made via MRI. Surgery is only performed on individuals with neurological impairment, and treatment is based on the clinical presentation. The preferred course of therapy is total surgical excision. We present a case that involves the successful surgical removal of an extradural spinal arachnoid cyst in a 10-year-old girl. Given the rarity of this pathology, its wide array of presenting symptoms, and the successful therapeutic protocol that was followed in this particular case, we believe this article shall prove beneficial to the medical fraternity.

## Introduction

Spinal arachnoid cysts account for a small proportion (approximately 1%) of all primary spinal mass lesions [1]. Extradural or intradural locations can both occur, with varying symptoms, depending on the site of the cyst. Extradural spinal arachnoid cysts are uncommon lesions whose pathophysiology is currently unknown. They are most frequently seen in the thoracic spine, followed by the lumbosacral and thoracolumbar areas [2]. They are characterized by a wide variety of clinical symptoms, ranging from asymptomatic lesions to severe myelopathy [3]. Due to the small number of cases that have been documented and the fact that a significant proportion of them have been asymptomatic, it is challenging to determine their incidence with accuracy. MRI is the preferred technique to evaluate this pathology [4]. In symptomatic instances, surgery is recommended, with a typically positive result [5]. We report a case characterized by a varied clinical picture and positive prognostic value and discuss the surgical therapy employed.

## Case presentation

**Figure 1:**
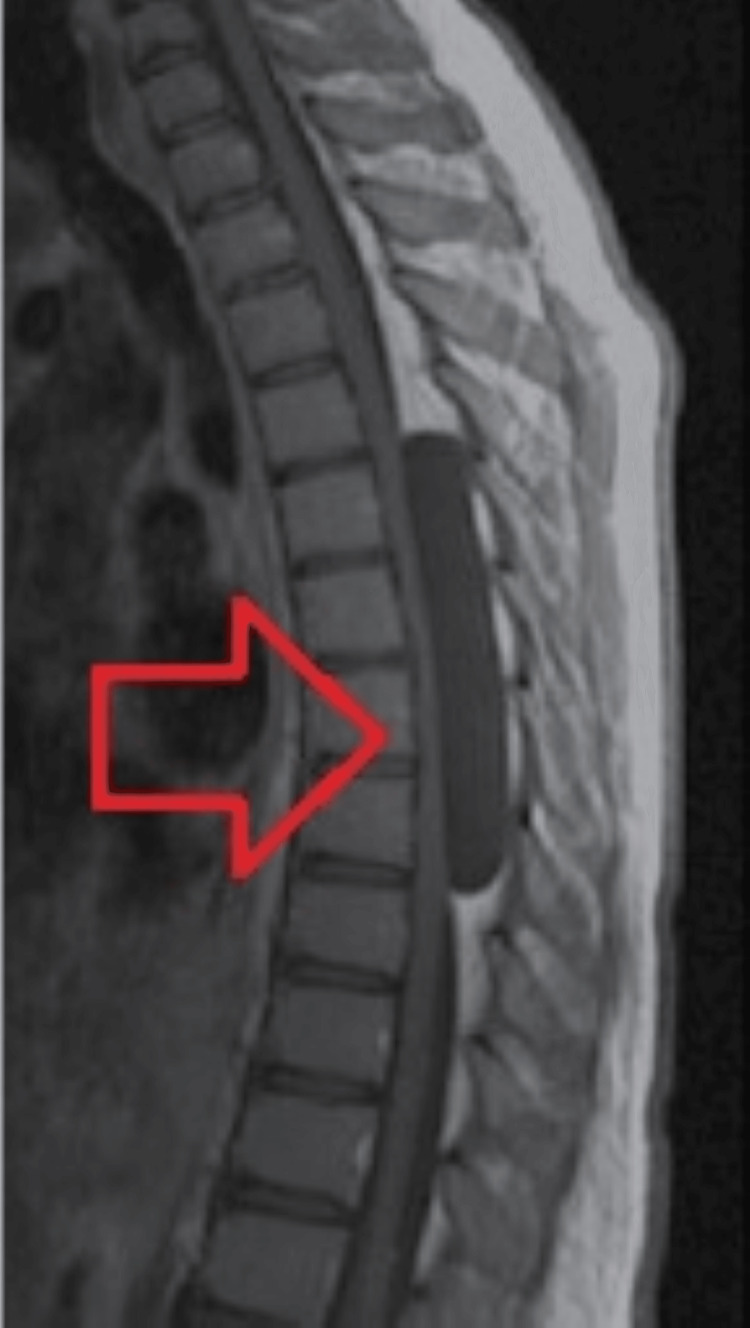
Thoracic T1-weighted MRI showing an extradural cyst MRI: magnetic resonance imaging

The patient was a 10-year-old girl who presented with progressive lower limb weakness, which had been present for eight months, along with paraesthesia and accompanying spasms. The patient had undergone some symptomatic relief regimens as well as a few physiotherapy sessions in order to manage the worsening limb weakness. There was no sphincteric dysfunction or back discomfort. At the time of her presentation, she was facing difficulty in carrying out her routine activities. The neurological evaluation revealed that the majority of the lower extremity muscles had power grade 3 spastic paraparesis. A cystic lesion squeezing the spinal cord posteriorly from the thoracic fourth (T4) to the thoracic eighth (T8) was visible on the MRI of the thoracic spine, as shown in Figure 1.

We removed the cyst completely and performed T4-T8 laminectomies. The pedicle was at the thoracic seventh (T7), dorsolateral to the nerve sleeve, which connected the cyst to the subarachnoid space. At the time of discharge, she had made exceptional postoperative progress and was able to walk alone. Twelve months following the operation, she was followed up at the outpatient clinic. The patient was kept under routine observation and neurological recovery following surgery was maintained, and all lower limb muscle groups were found to have grade 5 power.

## Discussion

Spinal extradural arachnoid cysts are a rare cause of myelopathy related to compression of the spinal cord [6]. The mechanism behind the origin of these cysts is currently unknown. Several hypotheses have been put forward regarding its etiology in the adult population: congenital; arachnoid adhesions secondary to an inflammatory process caused by virus, spirochetes, or bacteria; arachnoiditis secondary to subarachnoid hemorrhage, contrast media, spinal anesthetics, meningitis; traumatic injuries to the vertebral column; lumbar punctures used in diagnostic procedures; anesthetic and intradural surgery; and idiopathic causes [7]. It has been found that congenitally asymptomatic cysts can expand as a result of trauma and develop symptoms [8]. The expansion of spinal extradural arachnoid cysts may be influenced by pulsatile cerebrospinal fluid (CSF) dynamics, an osmotic gradient between the subarachnoid space and the cyst, and a valve-like mechanism between the cyst and subarachnoid space [3].

In most patients, spinal cysts of congenital origin appear in their adolescence or early adulthood, and the diverticulum often occurs in central areas, as was the case in our patient. The dorsal thoracic preference of spinal arachnoid extradural cyst, which was traditionally attributed to higher hydrostatic pressure from the CSF column, may also be explained by the congenital origin hypothesis [9]. The dorsal midline appears to be particularly vulnerable to maldevelopment since the neural tube grows from ventral to dorsal. Besides the dorsal preference, we also highlight here the thoracic placement by pointing out the spinal cord's more anterior position and the relative narrowing in comparison to the cervical and lumbar enlargements. Because of this, the dorsal thoracic spinal column's epidural potential space is larger and offers a higher possibility for cyst growth [10].

MRI is the preferred imaging technique for evaluation. The signal intensity of arachnoid cysts on all MRI sequences is identical to that of CSF [4]. Intradural arachnoid cyst is indicated by the inability to distinguish the cyst walls clearly. Arachnoid cysts may be distinguished from other spinal cysts, abscesses, and cystic tumors by using this imaging technique. When neurological symptoms arise as a result of cyst-induced spinal cord or nerve root compression, surgery is necessary [10,11]. Long-lasting discomfort and preoperative myelomalacia have both been linked to poor surgical outcomes. Total excision of the cyst is the most frequently recommended surgical procedure. However, the removal carries the risk of several consequences, including the cyst adhering to the nerve roots or the spinal cord, and may require the sacrifice of nerve roots [12].

## Conclusions

Spinal extradural cysts can lead to debilitating effects and early diagnosis and treatment are necessary for a favorable prognosis. The MRI has proven to be the gold standard imaging technique for investigation. Complete excision of the cyst, obliteration of the connecting pedicle, and watertight closure of the dural defect are the preferred surgical techniques to prevent recurrence. The surgical procedures, although complicated, considering the nerve plexus, can offer great relief to the patient if successful and result in favorable prognostic outcomes.
